# The frequency of Treg subsets distinguishes disease activity in ANCA vasculitis

**DOI:** 10.1002/cti2.1428

**Published:** 2022-11-11

**Authors:** Christian Agosto‐Burgos, Eveline Y Wu, Marie A Iannone, Yichun Hu, Susan L Hogan, Candace D Henderson, Kristin B Kennedy, Lauren Blazek, Carolina A Herrera, Dominique Munson, Ronald J Falk, Dominic J Ciavatta, Meghan E Free

**Affiliations:** ^1^ Division of Nephrology and Hypertension, Department of Medicine, UNC Kidney Center University of North Carolina at Chapel Hill Chapel Hill NC USA; ^2^ Department of Pathology and Laboratory Medicine University of North Carolina at Chapel Hill Chapel Hill NC USA; ^3^ Division of Pediatric Allergy, Immunology and Rheumatology, Department of Pediatrics University of North Carolina Chapel Hill NC USA; ^4^ Lineberger Comprehensive Cancer Center University of North Carolina at Chapel Hill Chapel Hill NC USA; ^5^ Department of Medicine University of North Carolina at Chapel Hill Chapel Hill NC USA; ^6^ Department of Genetics University of North Carolina at Chapel Hill Chapel Hill NC USA

**Keywords:** ANCA vasculitis, autoimmune disease, Tregs

## Abstract

**Objectives:**

T regulatory cells (Tregs) are a heterogeneous group of immunoregulatory cells that dampen self‐harming immune responses and prevent the development of autoimmune diseases. In anti‐neutrophil cytoplasmic autoantibody (ANCA) vasculitis, Tregs possess diminished suppressive capacity, which has been attributed to the expression of a FOXP3 splice‐variant lacking exon 2 in T cells (FOXP3Δ2 CD4^+^ T cells). However, the suppressive capacity of Tregs varies between subsets. We evaluated the frequency of Treg subsets in ANCA vasculitis as a potential explanation for diminished suppressive capacity.

**Methods:**

We developed a custom mass cytometry panel and performed deep immune profiling of Tregs in healthy controls, patients with active disease and in remission. Using these data, we performed multidimensional reduction and discriminant analysis to identify associations between Treg subsets and disease activity.

**Results:**

Total Tregs were expanded in ANCA vasculitis, which was associated with remission and the administration of rituximab and/or prednisone. The frequency of FOXP3Δ2 CD4^+^ T cells did not distinguish disease activity and this population had high expression levels of CD127 and lacked both CD25 and Helios, suggesting that they are not conventional Tregs. The frequency of CXCR3^+^, CD103^+^ and CCR7^+^ Tregs distinguished disease activity, and the combination of the frequency of these three Treg subsets segregated active patients from patients in remission and healthy controls. From these three subsets, the frequency of CXCR3^+^ Tregs distinguished patients with active disease with renal involvement.

**Conclusion:**

Treg heterogeneity can discriminate disease activity and should be explored as a biomarker of disease activity in ANCA vasculitis.

## Graphical abstract

The heterogeneity of the T regulatory cell (Treg) population and its implications in anti‐neutrophil cytoplasmic autoantibody (ANCA) vasculitis are unknown. Herein, we demonstrate that a combination of the frequency of CD103, CCR7 and CXCR3 on Tregs can discriminate disease activity. Treg heterogeneity should be explored as a biomarker of disease activity in ANCA vasculitis and other autoimmune diseases.

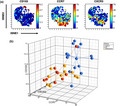

## Introduction

The primary function of T regulatory cells (Tregs) is to dampen self‐harming immune responses and to prevent the development of autoimmune diseases. However, altered functionality of Tregs has been reported in several autoimmune diseases, including ANCA vasculitis.^1^, ^2^, ^3^, ^4^ Although Tregs have previously been investigated as a homogenous subset of T cells in many studies, the Treg population is a highly heterogeneous population of immunoregulatory cells.^5^, ^6^, ^7^ Previous studies have established strong associations between specific Treg subsets and disease activity in other autoimmune diseases such as systemic lupus erythematosus (SLE) and rheumatoid arthritis (RA).^8^, ^9^, ^10^ For example, the frequency of Helios^+^ Tregs positively correlates with disease activity, but the frequency of CD45RA^−^FOXP3^low^ Tregs correlates negatively with disease activity in SLE patients.^8^, ^10^ In the context of RA, a positive correlation was observed between the frequency of a subset of Tregs expressing the Th17 transcription factor RORyt and the disease activity score.^9^ This suggests that Treg biology could be exploited as a biomarker of disease activity.

Previous studies regarding Tregs in ANCA vasculitis were performed using bulk Tregs.^1^, ^2^, ^3^, ^4^ While it is certain that Tregs have diminished suppressive capacity in ANCA vasculitis and the expression of a FOXP3 variant lacking exon 2 in CD4^+^ T cells is negatively correlated with the suppressive capacity of Tregs, it remains unclear which subset or subsets of Tregs are contributing to the impaired phenotype.^3^ Diminished Treg suppressive capacity is usually attributed to intrinsic Treg defects. However, it is well established that the suppressive capacity of Tregs varies between subsets.^11^, ^12^, ^13^ Changes in the proportion of Treg subsets could be an underlying driver of the diminished suppressive capacity of Tregs reported in ANCA vasculitis. To understand the Treg population in the circulation of ANCA vasculitis patients on a granular level, we developed a custom mass cytometry panel consisting of 28 markers. We hypothesised that changes in the frequency of Treg subsets would distinguish disease activity and organ involvement in ANCA vasculitis.

Furthermore, current treatments for ANCA vasculitis rely on the administration of immunosuppressive drugs with adverse side effects.^14^, ^15^, ^16^ Infusion of functional and stable autologous expanded Tregs could induce sustained long‐term remission in patients with autoimmune disease and reduce the adverse side effects of current immunosuppressive treatments.^17^, ^18^ To exploit Tregs as a biomarker and therapeutic approach in ANCA vasculitis, a strong foundation and understanding of Treg heterogeneity and their implications in disease manifestation is imperative.^19^, ^20^

## Results

### Bulk Tregs

**Figure 1:**
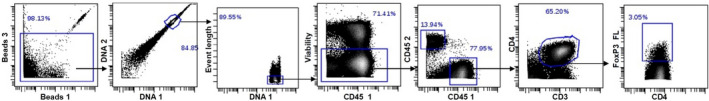
Mass cytometry gating strategy: Tregs were defined as live CD3^+^CD4^+^FOXP3^+^ after single and live cell discrimination.

**Figure 2:**
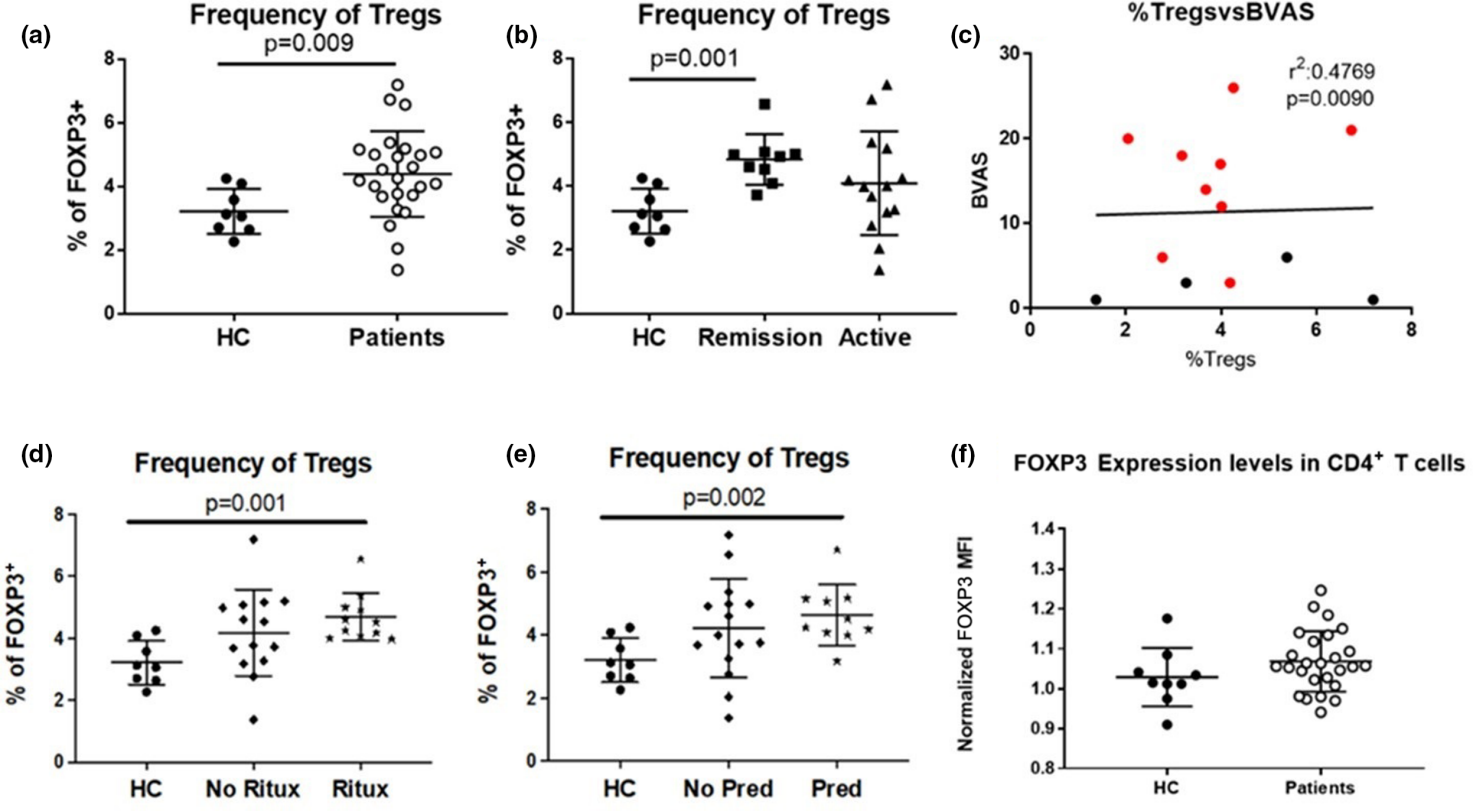
The expansion of Tregs in anti‐neutrophil cytoplasmic autoantibody (ANCA) vasculitis is associated with remission and the administration of immunosuppressive drugs. **(a)** Frequency of Tregs from CD4^+^ T cells in the peripheral circulation of HCs; closed circle (*n* = 8) and patients; open circle (*n* = 25). **(b)** Frequency of Tregs from CD4^+^ T cells in the peripheral circulation of HCs; closed circle (*n* = 8) and patients in remission; closed square (*n* = 9) and with active disease; closed triangle (*n* = 14). **(c)** Correlation between the frequency of Tregs and Birmingham Vasculitis Activity Score (BVAS). **(d)** Frequency of Tregs from CD4^+^ T cells in the peripheral circulation of HCs (*n* = 8), patients that did not receive rituximab (no Ritux; *n* = 14); closed diamond and patients that received rituximab; closed star (Ritux; *n* = 11) at the time of sample collection or 6 months prior. **(e)** Frequency of Tregs from CD4^+^ T cells in the peripheral circulation of HCs (*n* = 8), patients that did not receive prednisone; closed diamond (no Pred; *n* = 15) and patients that received prednisone; closed star (Pred; *n* = 10) at the time of sample collection or 6 months prior. **(f)** Normalised FOXP3 protein expression levels in peripheral Tregs from HCs (*n* = 8) and patients (*n* = 25). Bars shown are median and interquartile range.

To examine Treg heterogeneity in patients with ANCA vasculitis, we developed a custom mass cytometry panel consisting of 28 markers (chemokine and cytokine receptors, migration, adhesion and activation molecules, membrane‐bound cytokines and immune‐checkpoint receptors/ligands; Supplementary table 1). All the markers for this panel were chosen after a thorough literature review to identify markers of Treg subsets. At the time of inception of this study, mass cytometry allowed us to overcome the current limitations of conventional flow cytometry, which could only examine a few markers simultaneously because of the limitations of spectral overlap and spillover. Tregs were defined holistically as CD4^+^FOXP3^+^ T cells using our mass cytometry gating strategy (Figure 1). We first measured the frequency of bulk Tregs, defined as CD4^+^ FOXP3^+^, in ANCA vasculitis patients and healthy controls (HCs) (Figure 2a). Similar to previous studies, we found that bulk Tregs are expanded in ANCA vasculitis patients compared to HCs (Figure 2b).^2^, ^3^, ^21^ We found that patients in remission have an increased frequency of total Tregs compared to HC and patients with active disease (Figure 2c). No correlation was observed between the frequency of total Tregs and Birmingham Vasculitis Activity Score (BVAS; Figure 2d). This suggests that patients in remission predominantly account for the expansion of Tregs seen in ANCA vasculitis patients.

Previous studies have shown that rituximab and prednisone treatment can lead to an increased frequency of Tregs in SLE patients.^22^, ^23^, ^24^, ^25^, ^26^ Therefore, we examined whether the administration of rituximab and prednisone at the time of sample collection or 6 months prior could be linked to the expansion of Tregs in ANCA vasculitis. Our data demonstrate that Tregs are expanded in patients that received rituximab (Figure 2e) and prednisone (Figure 2f). Similar to other reports, our data demonstrate that Tregs from ANCA vasculitis patients and healthy controls express similar protein levels of FOXP3 (Figure 2g).^4^ Taken together, these data suggest that the frequency of bulk Tregs can distinguish remission during ANCA vasculitis. However, changes in specific Treg subsets may be of more importance than the frequency of total Tregs. To this end, we decided to measure the frequency of several subsets of Tregs.

### FOXP3Δ2 CD4 ^+^ T cells

**Figure 3:**
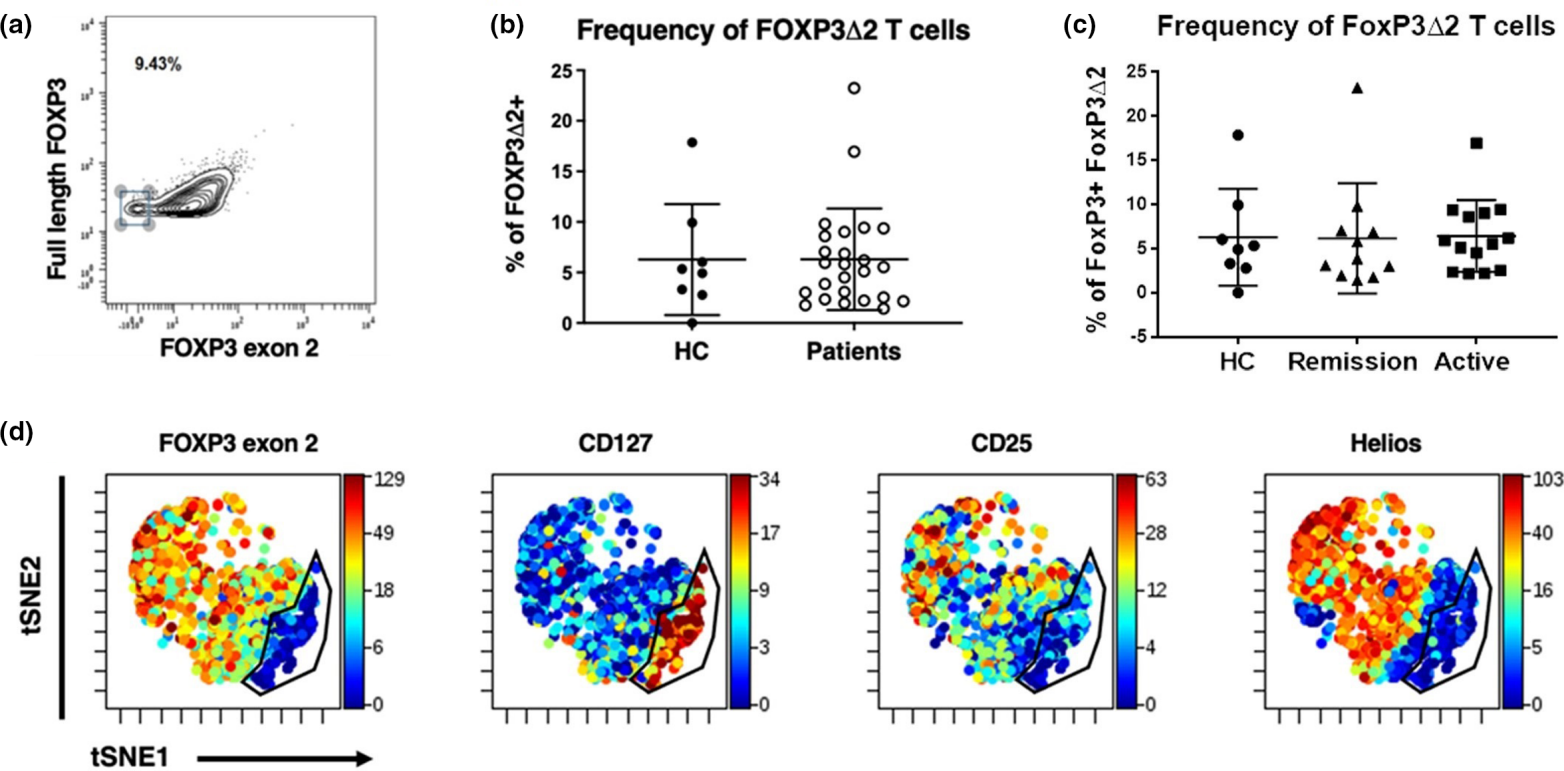
FOXP3Δ2 CD4^+^ T cells are not conventional (CD25^high^CD127^−/low^) Tregs and cannot distinguish disease activity in anti‐neutrophil cytoplasmic autoantibody (ANCA) vasculitis. **(a)** Representative mass cytometry gate for the detection of FOXP3Δ2 CD4^+^ T cells from total FOXP3^+^CD4^+^ T cells. **(b)** Frequency of FOXP3^+^CD4^+^ T cells from CD4^+^ T cells in the peripheral circulation of HCs (*n* = 8) and patients (*n* = 25). **(c)** Frequency of peripheral FoxP3Δ2 CD4^+^ T cells from FOXP3^+^CD4^+^ T cells in HCs (*n* = 8) and patients in remission (*n* = 9) and with active disease (*n* = 13). **(d)** VisNE analysis of FOXP3^+^CD4^+^ T revealing the expression levels of FOXP3 exon 2, CD127, CD25 and Helios (from left to right) in FoxP3Δ2 CD4^+^ T cells. Bars shown are median and interquartile range.

Tregs' diminished suppressive capacity is more pronounced during active disease, and the frequency of a CD4^+^ T cell population expressing a variant of FOXP3 lacking exon 2 (FOXP3Δ2 CD4^+^ T cells) has been reported to be negatively correlated with Treg suppressive capacity in ANCA vasculitis. Therefore, we examined whether changes in the frequency of a population of CD4^+^ T cells expressing this variant could distinguish disease activity in ANCA vasculitis. To this end, we utilised an antibody against exon 2 of FOXP3 and an antibody against the N terminus of FOXP3 to detect and measure the frequency of FOXP3Δ2 CD4^+^ T cells (Figure 3a). Our data demonstrate that ANCA vasculitis patients and HCs possess similar frequencies of FOXP3Δ2 CD4^+^ T cells (Figure 3b). We also found that patients with active disease and in remission possess similar frequency levels of FOXP3Δ2 CD4^+^ T cells (Figure 3c), which is consistent with previous reports.^27^ Although FOXP3 is a transcription factor required for Treg identity, stability and functionality, it is not uniquely expressed in Tregs. Therefore, we determined whether FOXP3Δ2 CD4^+^ T cells are conventional Tregs based on the expression levels of CD25, CD127 and Helios. We performed a VisNE clustering analysis on CD4^+^FOXP3^+^ cells. We found that FOXP3Δ2 CD4^+^ T cells are characterised by high expression levels of CD127 and lack expression of both CD25 and Helios (Figure 2d). Consistent with this result, unbiased VisNE analysis on total CD4^+^ T cells revealed that the expression of FOXP3Δ2 was detected in CD4^+^ T cells expressing low or intermediate levels of FOXP3 in both HCs and patients (Figure 3b). In contrast, expression of full‐length FOXP3 containing exon was confined to a population of CD4^+^ T cells with high levels of FOXP3 (Supplementary figure 1).

### CD103 ^+^, CCR7 ^+^ and CXCR3 ^+^ Treg subsets

**Figure 4:**
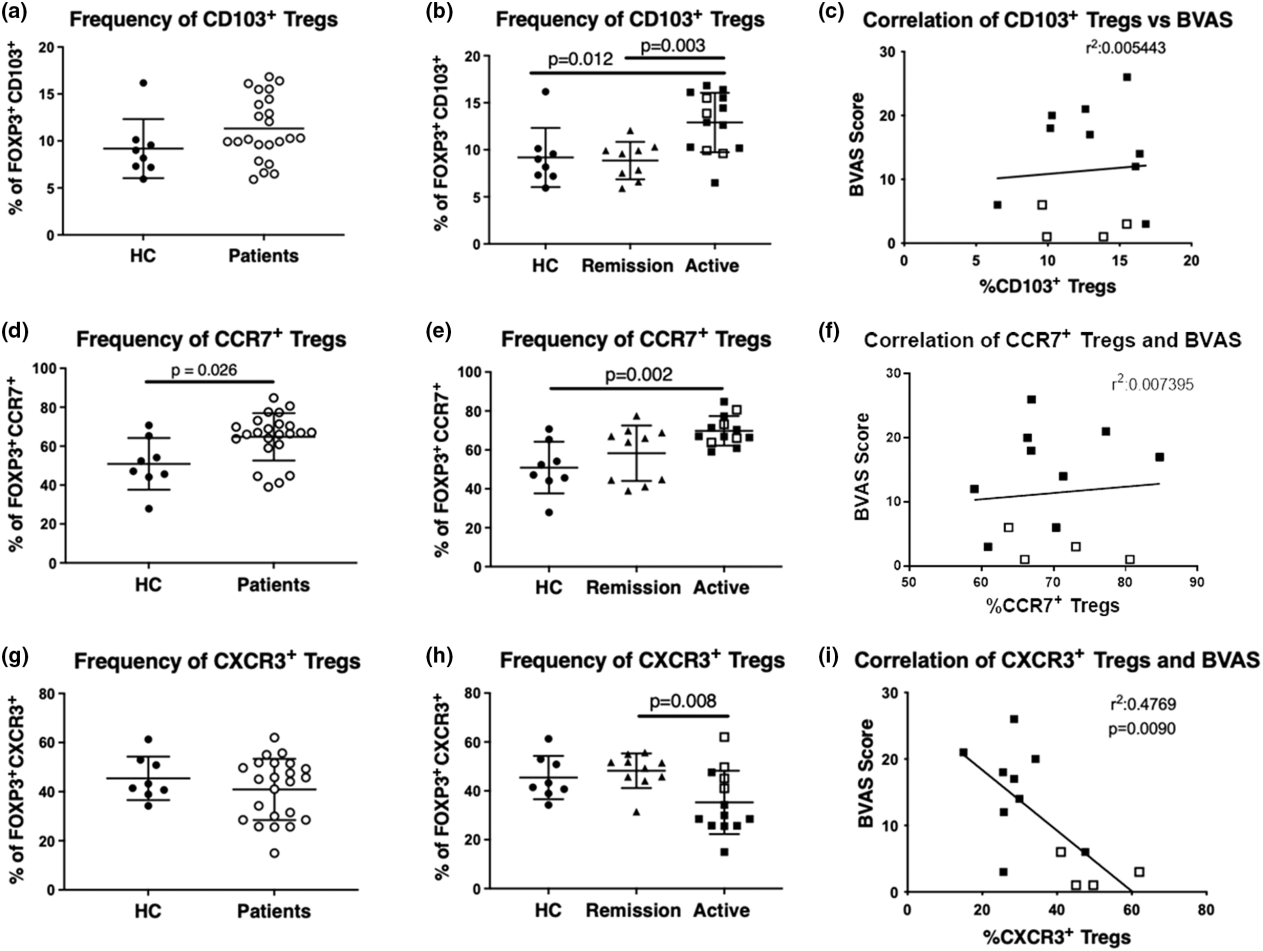
Changes in the frequency of CD103^+^, CCR7^+^ and CXCR3^+^ Tregs distinguish disease activity in anti‐neutrophil cytoplasmic autoantibody (ANCA) vasculitis. **(a, d, g)** Frequency of CD103^+^, CCR7^+^ and CXCR3^+^ Tregs in the peripheral circulation of HCs (*n* = 8) and patients (*n* = 23). **(b, e, h)** Frequency of CD103^+^, CCR7^+^ and CXCR3^+^ Tregs from CD4^+^ T cells in the peripheral circulation in HCs (*n* = 8) and patients in remission (*n* = 10) and with active disease (*n* = 13). **(c, f, i)** Correlation between the frequency of CD103^+^, CCR7^+^ and CXCR3^+^ Tregs and Birmingham Vasculitis Activity Score (BVAS). Bars shown are median and interquartile range. Patients with active disease with renal involvement are depicted in closed squares. Correlation's significance was determined using the non‐parametric Spearman's correlation test.

Since FOXP3Δ2 CD4^+^ T cells, which are not conventional Tregs, do not distinguish disease activity in ANCA vasculitis, we examined whether the frequency of other Treg subsets might distinguish disease activity in ANCA vasculitis. Interestingly, ANCA vasculitis patients have an increased frequency of CCR7^+^ Tregs (Figure 4d), but not CD103^+^ (Figure 4a) and CXCR3^+^ (Figure 4g) Tregs. Additional analysis examined Treg subset frequencies as they relate to patient disease serology (MPO‐ANCA vs. PR3‐ANCA) and found that serology did not significantly impact Treg subset frequencies (Supplementary figure 3). However, examining the data based on disease activity (active disease vs. remission) did impact Treg subset frequencies. We found that both CD103^+^ (Figure 4b) and CCR7^+^ (Figure 4e) Treg subsets are expanded in active disease compared to patients in remission and/or healthy controls. In contrast, we found that CXCR3^+^ Tregs are decreased during active disease compared to remission and healthy controls (Figure 4f). Because BVAS is heavily influenced by renal involvement, we tested whether the frequency of CD103^+^, CCR7^+^ and CXCR3^+^ Tregs was associated with patients with active renal disease (Figure 4g–i). We found that the frequency of CXCR3^+^ Tregs, but not CD103^+^ and CCR7^+^ Tregs, tended to be different between patients with renal involvement vs. patients with non‐renal involvement during active disease. This suggests that renal involvement is associated with the frequency of CXCR3^+^ Tregs, but not CD103^+^ and CCR7^+^ Tregs. Correlation analysis with BVAS supports the association between CXCR3^+^ Tregs, but not CD103^+^ and CCR7^+^ Tregs with renal involvement (Figure 4c, f and i). Furthermore, we found that the frequency of CXCR3^+^ Tregs negatively correlates with BVAS (Figure 4i). Through this analysis, we determined that the frequency of CXCR3^+^ Tregs can distinguish patients who have active disease with renal involvement versus patients with active disease with non‐renal involvement (Figure 4h and i).

### Discriminant analysis

**Figure 5:**
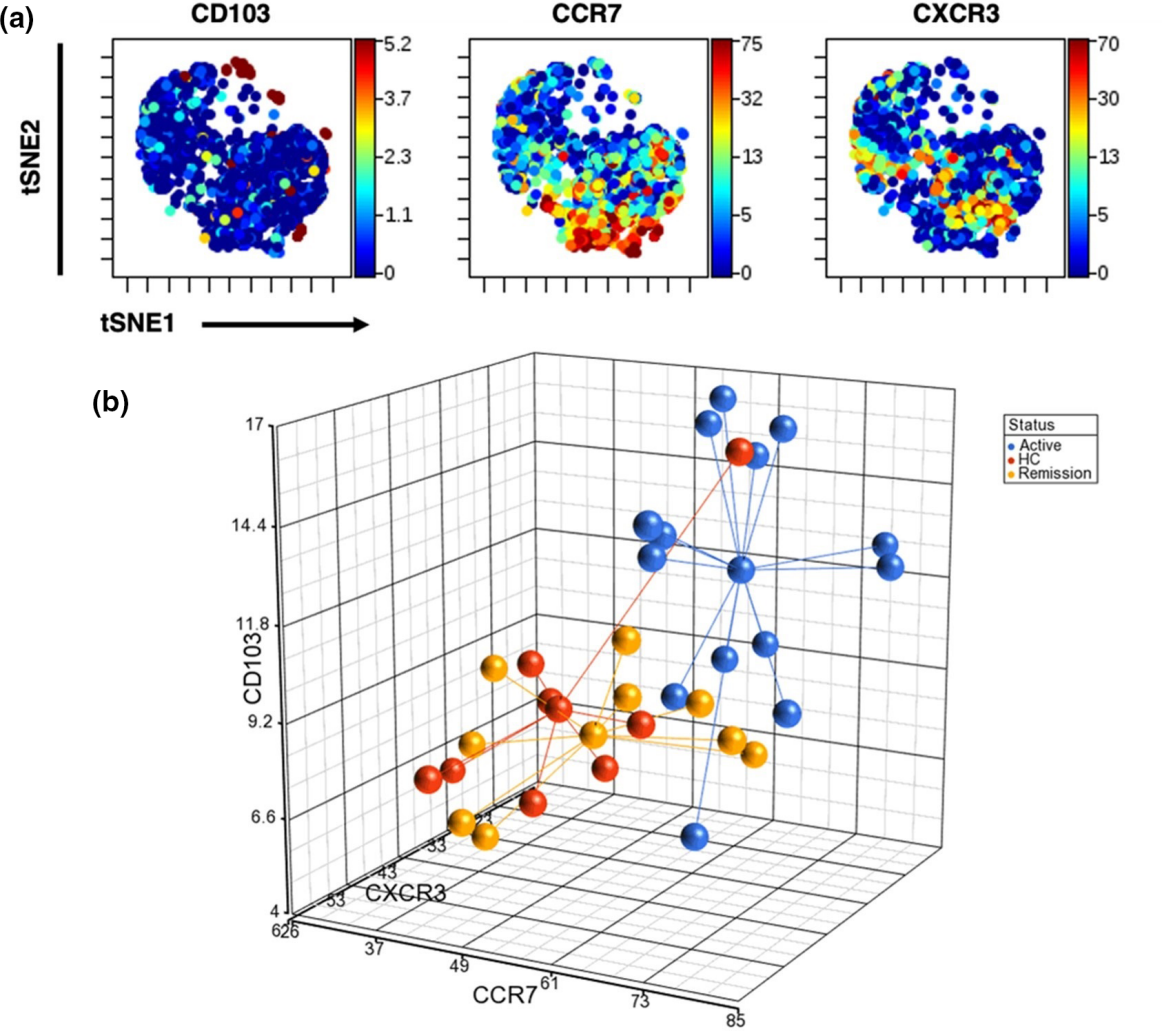
A combination of the frequencies of CD103^+^, CCR7^+^ and CXCR3^+^ Tregs segregates active patients from patients in remission and healthy controls. **(a)** VisNE analysis revealing clusters of CD103^+^, CCR7^+^ and CXCR3^+^ Tregs (left to right). **(b)** Three‐dimensional plot showing the segregation of active patients (blue) from patients in remission (yellow) and healthy controls (orange) when the frequencies of CD103^+^, CCR7^+^ and CXCR3^+^ Tregs are combined.

We found that three subsets of Tregs can distinguish disease activity in ANCA vasculitis when examined on an individual subset basis. However, it remains unknown whether the expression of CD103, CCR7 and CXCR3 are co‐expressed and highlight the same subsets of Tregs. Therefore, we performed an unbiased clustering analysis of Tregs using VisNE and found that the expression of CD103, CCR7 and CXCR3 is predominantly mutually exclusive (Figure 5a). This suggests that these markers identify three individual subsets of Tregs.

We then determined whether a combination of the frequency of CD103, CCR7 and CXCR3 segregates active patients from patients in remission and HCs. Therefore, we combined the frequency of CD103^+^, CCR7^+^ and CXCR3^+^ Tregs for each sample and plotted the values in a tridimensional graph (Figure 5b). We found that combining the frequency of the CD103^+^, CCR7^+^ and CXCR3^+^ Tregs better segregates patients with active disease from patients in remission and healthy controls (Figure 5b). Consistent with this, the discriminant analysis revealed segregation between patients with active disease from those in remission and from healthy controls using the frequency of all three subsets (Supplementary table 2). The segregation was primarily driven by the frequency of CCR7^+^ and CXCR3^+^ Tregs as evidenced by the total error rates; 22% for all three subsets, 38% for CCR7^+^ and CXCR3^+^ Tregs, 50% for CD103^+^ and CXCR3^+^ Tregs and 50% for CD103^+^ and CXCR3^+^ Tregs (Supplementary table 2). Taken together, this suggests that by combining the frequency of the CD103^+^ and CCR7^+^, Tregs better segregates patients with active disease from patients in remission and healthy controls.

## Discussion

This is the first study to examine Treg heterogeneity in the context of ANCA vasculitis. Using our custom mass cytometry panel, we profiled Tregs and measured the frequency of total FOXP3^+^ Tregs and several subsets of Tregs in healthy controls, patients in remission and patients with active disease. We found that total Tregs (CD4^+^ FOXP3^+^) are increased in ANCA vasculitis, and it is primarily driven by patients in remission and associated with rituximab and prednisone administration. Here, we show that FOXP3Δ2 CD4^+^ T cells, which were previously identified to correlate with Treg's suppressive capacity in ANCA vasculitis, are not conventional (CD25^high^CD127^−/low^) Tregs, and their frequency does not distinguish disease activity.^2^ Interestingly, we identified novel associations between the frequency of the CD103^+^, CCR7^+^ and CXCR3^+^ Treg subsets and disease activity and organ involvement in active disease. Both CD103^+^ and CCR7^+^ Tregs are increased in patients with active disease. In contrast, CXCR3^+^ Tregs are reduced during active disease in patients with renal involvement. We also demonstrated that a combination of the frequency of CD103^+^, CCR7^+^ and CXCR3^+^ Tregs better segregates patients with active disease from patients in remission and healthy donors, suggesting that Treg heterogeneity has the potential to become a biomarker of disease activity.

Using our mass cytometry approach, we observed an expansion of bulk Tregs in our patient cohort. This is consistent with other reports in which Tregs increase in ANCA vasculitis patients.^2^, ^3^, ^21^, ^28^ Similar to other reports, this expansion seems to be primarily driven by patients in remission.^27^ We observed an expansion of Tregs in patients that received rituximab and prednisone. This suggests that immunosuppressive treatments could lead to the expansion of Tregs seen in patients with ANCA vasculitis. However, rituximab and prednisone treatment do not entirely explain the expansion of Tregs since some untreated patients also presented an expansion of Tregs. Therefore, a current limitation is deciphering whether medication administration is causative or merely associative with the disease status when analysing Treg subsets. Perhaps, disease manifestation could also contribute to this expansion since it has been previously reported that an increase in the frequency of Tregs is associated with a shorter time to clinical remission.^4^ However, studies in a mouse model of MPO‐ANCA‐induced glomerulonephritis sought to elucidate the relationship between anti‐CD20‐induced B‐cell depletion and T cells. This study was able to demonstrate that antigen‐specific Tregs could be induced by splenic macrophages processing apoptotic B cells.^29^ The removal and processing of the apoptotic B cells by splenic macrophages likely results in the release of immunomodulatory cytokines that positively influence the Treg pool. In addition, it has also been reported in some studies that Tregs are decreased in ANCA vasculitis.^4^ The discrepancies as to whether Tregs are increased or decreased during ANCA vasculitis could be partially explained by differences in rituximab and prednisone treatment between cohorts. Taken together, this suggests that an expansion of partially functional Tregs could compensate for their decreased overall function and be sufficient to induce remission in patients. In addition, the expansion of Tregs in patients in remission suggests that infusion of *ex‐vivo* expanded and functional, autologous Tregs could be an alternative treatment to induce or maintain remission in ANCA vasculitis.

Although it is clear that Tregs have impaired suppressive capacity in ANCA vasculitis, little is known about the underlying drivers of this impairment. The only evidence of a potential driver of Tregs dysfunctionality in ANCA vasculitis comes from a study, in which the frequency of FOXP3Δ2 CD4^+^ T cells negatively correlated with Tregs' suppressive capacity.^3^ In that study, conventional Tregs were isolated using flow activated cell sorting based on their surface expression levels of CD25 and CD127 and defined as CD25^high^CD127^−/low^. CD25^high^CD127^−/low^ Tregs were then co‐cultured with activated autologous and non‐autologous T effector cells to assess their suppressive capacity.^3^ However, we demonstrate that FOXP3Δ2 CD4^+^ T cells are CD25^low^CD127^high^, suggesting that FOXP3Δ2 CD4^+^ T cells were not present within the sorted CD25^high^CD127^−/low^ Tregs and are less likely a driver of the impaired suppressive capacity of Tregs observed in ANCA vasculitis.^3^ It has also been reported that FOXP3Δ2 CD4^+^ T cells are increased in ANCA vasculitis patients.^3^ However, we did not observe an expansion of FOXP3Δ2 CD4^+^ T cells in our cohort. Similar to other reports, we also demonstrate that the frequency of FOXP3Δ2 CD4^+^ T cells does not distinguish disease activity during ANCA vasculitis.^27^ In addition, the lack of expression of Helios suggests that FOXP3Δ2 CD4^+^ T cells are not thymic‐derived Tregs.^30^ It is well reported in the literature that activated CD4^+^ T cells can upregulate the expression of FOXP3 transiently.^31^, ^32^ Perhaps, FOXP3Δ2 CD4^+^ T cells are activated effector T cells that transiently upregulated the expression of FOXP3, which can explain their diminished suppressive capacity and Th17 pro‐inflammatory cytokine profile.^33^ Furthermore, it has been documented that other splice variants of FOXP3 are upregulated upon activation.^34^ Taken together, this suggests that FOXP3Δ2 CD4^+^ T cells are not conventional Tregs and that the expression of this variant is unlikely the driver of Tregs' impaired suppressive capacity in ANCA vasculitis.

This study demonstrates novel correlations between the frequency of CXCR3^+^, CD103^+^ and CCR7^+^ Tregs and disease activity, and organ involvement in ANCA vasculitis. We observed an expansion of CCR7^+^ Tregs, which have been previously reported to suppress the proliferation of antigen‐activated T cells and to preferentially home in antigen‐draining lymph nodes where they dampen inflammatory responses.^35^, ^36^ CCR7 deficiency in mice leads to spontaneous signs of chronic autoimmune kidney disease and IgG deposition in the glomeruli.^37^, ^38^ Furthermore, the adoptive transfer of WT Tregs into CCR7KO mice protects CCR7KO mice from developing acute nephritis.^38^ Although CCR7^+^ Tregs provide protection against renal autoimmunity, they do not home to the kidneys, which may explain why we did not see an association between their frequency and active renal disease.^36^, ^38^ It could be also possible that the frequency of CCR7^+^ Tregs is increased in all forms of active disease. Taken together, this suggests that CCR7^+^ Tregs are pivotal for ameliorating renal autoimmune diseases and that they might play a significant role in promoting disease remission in ANCA vasculitis. Further studies need to dissect the involvement of CCR7^+^ Tregs in the context of ANCA vasculitis. We also observed an expansion of CD103^+^ Tregs in active patients. However, little is known about the role of CD103^+^ Tregs in humans because of their minor frequency, which also makes them a less likely therapeutic target.^39^, ^40^, ^41^ Herein, we demonstrate that CXCR3^+^ Tregs are decreased in ANCA vasculitis patients with active renal disease. CXCR3^+^ Tregs have been previously shown to be enriched and recruited into the kidneys of ANCA vasculitis patients owing to the expression of CXCR3's ligands, CXCL10 and CXCL9, in renal tissue.^42^ This may suggest that the decrease in the frequency of CXCR3^+^ Tregs that we are reporting in the periphery of patients could be the consequence of the migration of CXCR3^+^ Tregs into the kidneys, and not because of a reduction in their generation. Interestingly, deletion of CXCR3^+^ in Tregs leads to aggravated glomerulonephritis in a murine model of nephrotoxic glomerulonephritis.^42^ In addition, we also saw some trends, although not significant, between the administration of rituximab and prednisone and the frequency of CXCR3^+^, CD103^+^ and CCR7^+^ Tregs. However, it remains unclear whether these trends are a consequence of the immunosuppressive treatment or active disease since ~ 60% of the treated patients were also active patients (Supplementary figure 1). Taken together, this suggests that changes in the frequency of Treg subsets could potentially contribute to the diminished suppressive capacity of total Tregs reported in ANCA vasculitis, specifically during active disease.^1^ Furthermore, CXCR3^+^ Tregs are potentially important for ameliorating glomerulonephritis during ANCA vasculitis. Infusion of *ex‐vivo* expanded CXCR3^+^ Tregs could become a precision medicine treatment for those patients that suffer from renal ANCA vasculitis.

One of the limitations of this study is the lack of longitudinal samples. With the advent of new flow cytometry technologies (Cytek Aurora) with far larger capacities of fluorophore detection, future studies could examine Treg heterogeneity longitudinally and isolate by flow cytometry sorting unique Treg subsets. Although it is evident in this study that the frequency of CXCR3^+^, CD103^+^ and CCR7^+^ Tregs changes cross‐sectionally during active disease, it remains still unknown whether the frequency of these Tregs subsets also changes longitudinally between remission and relapse. However, since it is estimated that around 50% of patients relapse 5 years after the induction of remission, it was challenging to obtain relapse–remission paired samples for this study.^43^ Further studies need to be developed to address this question. Additionally, Treg plasticity is increasingly a concern in the field of autoimmunity. Pro‐inflammatory milieus can skew Tregs into effector T cells which would not only impact the frequencies of Tregs but also increase inflammatory effector subsets. This has recently been documented in RA wherein Tregs not only exhibit decreased suppressive capacity but are also prone to skewing into Th17 effector cells.^44^ Various cytokine milieus are more likely to alter Treg functionality than others, as IL‐6 can promote FOXP3^+^ cells to become Th17 cells.^45^ What remains unanswered is whether certain Treg subsets, such as those identified in this study, are more or less susceptible to phenotypic skewing. Another limitation of these findings is the lack of functional data assessing the suppressive capacity of CXCR3^+^ and CCR7^+^ Tregs in patients because of the low percentage of cells in peripheral blood samples. This poses a challenge when developing and executing functional assays with the appropriate experimental design and controls to assess the suppressive capacity of these populations. In addition, all the data shown here represent what is occurring in the peripheral circulation of patients, which may or may not represent what is happening in the sites of inflammation during human ANCA vasculitis. Lastly, since many of the active patients were treated with rituximab and/or prednisone, it remains unknown to what degree immunosuppressive treatment has any effect on the frequency of the Treg subsets reported here.

This is the first study identifying associations between the frequency of specific subsets of Tregs and their possible implications in disease activity and organ involvement in ANCA vasculitis. The findings of this study will help set the foundation for the development of tailored Treg‐based therapies for ANCA vasculitis.

## Methods

### Human subjects

Patients with ANCA vasculitis and healthy controls were recruited, gave informed written consent and participated according to the guidelines of the University of North Carolina Office of Human Research Ethics/Institutional Review Board (IRB study #97–0523). Patients were diagnosed according to the Chapel Hill Consensus Conference. Disease activity was assigned based on BVAS and chart review covering 3 months before and after the sample date. Active disease was defined as a BVAS ≥ 3 with clinical and/or laboratory evidence of disease. Remission was defined as a BVAS of 0 and no clinical or laboratory evidence of disease activity within 3 months of the sample. Patients with unclear disease activity were excluded from analysis related to disease activity. Patient and HC demographics are summarised in Table 1. Patients with drug‐induced forms of ANCA and overlapping disease and infections were excluded.

**Table 1:** Demographics of ANCA vasculitis patients and healthy controls

Characteristics		HC	Patients
Sample size (*n*)		8	25
Age	Mean ± SD	52 ± 15	61 ± 15
	Median (IQR)	49 (45,62)	64 (51,71)
Sex	Female	4 (50%)	14 (56%)
	Male	4 (50%)	11 (44%)
Ethnicity	Caucasian	7 (88%)	21 (84%)
	Black	0 (0%)	3 (12%)
	Hispanic/Latino	1 (12%)	1 (4%)
Serotype	MPO		13 (52%)
	PR3		11 (44%)
	Neg		1 (4%)
Diagnosis	MPA		12 (48%)
	GPA		11 (44%)
	Renal Limited		2 (8%)
Activity status	Remission		10 (40%)
	Active		13 (52%)
	Unclear		2 (8%)
eGFR (Mean ± SD)	Remission		46.7 ± 15.0
	Active		43.8 ± 26.8
	Unclear		13.0 ± 2.8
Serum creatine (Mean ± SD)	Remission		2.2 ± 1.0
	Active		1.4 ± 1.8
	Unclear		3.4 ± 0.4
Organ Involvement in active patients	Systemic		6 (46%)
	Cutaneous		4 (30%)
	Ear, nose, and throat		2 (15%)
	Renal		10 (76%)
	Nervous System		0 (0%)
	Chest		4 (30%)
	Abdominal		0 (0%)
	Cardiovascular		0 (0%)
	Mucous membranes/Eyes		1 (7%)
Medications History up to 6 months prior to sample collection	Rituximab		10 (38%)
	Prednisone		10 (38%)
	Solumedrol		8 (30%)
	Cyclophosphamide		4 (15%)
	Plasmapheresis		3 (11%)
	Myfortic		1 (3%)
	Cellcept		1 (3%)
	Azathioprine		1 (3%)
	Plaquenil		1 (3%)

ANCA, anti‐neutrophil cytoplasmic autoantibody; GPA, granulomatosis with polyangiitis; HC, healthy controls; IQR, interquartile range; Lim, limited‐renal small vessel vasculitis; MPA, microscopic polyangiitis; MPO, myeloperoxidase; PR3, proteinase 3; SD, standard deviation; Unclear; unable to assign activity status after chart review.

### Peripheral blood mononuclear cell isolation and CD4+ T cells enrichment

Blood from patients and healthy controls were collected using BD Vacutainer^©^ CPT™ Mononuclear Cell Preparation Tubes (BD Biosciences; Cat.362761) and spun at 1650 RCF for 15 min to isolate peripheral blood mononuclear cells (PBMCs). PBMCs were isolated from blood samples from patients and healthy control after density centrifugation. Cells were then washed twice with 1× PBS (Gibco^©^, Cat. 10010023) at 500 *g* for 5 min. PBMCs were resuspended in 10 million cells per mL in freezing media (FBS + 10% DMSO; Sigma‐Aldrich, Cat. D2650‐5X5ML) and cryopreserved. Cryopreserved PBMCs were thawed at 37°C, and washed twice with 1x PBS at 500 *g* for 5 min. As per the manufacturer's protocol specifications, CD4^+^ T cells were then isolated from PBMCs using an EasySep™ Human CD4^+^ T cells enrichment kit (STEMCELL™, Cat. 19052). Isolated CD4^+^ T cells were washed twice with 1× carrier‐free PBS (Rockland, Cat. MB‐008) and resuspended in 1–2 million cells per mL for staining.

### Mass cytometry staining, acquisition and analysis

CD4^+^ T cells were isolated from cryopreserved PBMCs and stained with a custom antibody cocktail containing 28 markers (see Supplementary table 1). Antibodies were optimised and validated using a Helios™ mass cytometer (Fluidigm^®^). When indicated, carrier‐free antibodies were purchased from several companies and conjugated with metal isotypes using a Multi‐metal Maxpar^®^ labelling kit (Fluidigm^®^) according to the manufacturer's protocols. Conjugations were performed either in‐house, by the Leeder Lab (CyTOF^®^ Core) at Harvard University or by Fluidigm^®^. The same spike‐in control was used throughout all of the mass cytometry runs for data quality, reproducibility and rigour.

A total of 1–2 million CD4^+^ T cells were stained with 1 μm Cell‐ID™ Cisplatin 195 (Fluidigm^®^, Cat. 201 064) in 1× carrier‐free PBS (Rockland, Cat. MB‐008) for 5 min at room temperature. Cells were then washed twice using Maxpar^®^ Cell Staining Buffer (Fluidigm^®^, Cat. 201 068) for 10 min at 500 *g*. Cells were resuspended in 1–2 million cells per 50 μL in Maxpar^®^ Cell Staining Buffer (Fluidigm^®^, Cat. 201 068) and stained with 50 μL of a surface antibody cocktail for 30 min at room temperature. CD4^+^ T cells were washed twice using Maxpar^®^ Cell Staining Buffer (Fluidigm^®^, Cat. 201 068) for 10 min at 500 *g*. Cells were then fixed with 2% Pierce™ 16% Formaldehyde (Thermo Scientific™, Cat. 28 906) for 1 h at room temperature. To detect intracellular markers, cells were permeabilised and stained using the eBioscience™ Foxp3/Transcription Factor Staining Buffer Set (Invitrogen™, Cat. 00–5523‐00) according to the manufacturer's recommendations. CD4^+^ T cells were then stained with Cell‐ID™ Intercalator‐Ir 125 μm (Fluidigm^®^, Cat. 201192A) diluted in Maxpar^®^ Fix & Perm Buffer (Fluidigm^®^, Cat. 201 067) overnight at 4°C. Cells were then washed twice with Maxpar^©^ Cell Staining Buffer (Fluidigm^®^, Cat. 201 068) for 10 min at 500 *g* and resuspended in 0.5 million cells per mL in Maxpar^®^ Cell Acquisition Solution Plus for CyTOF^®^ XT (Fluidigm^®^, Cat. 201 244). For runs quality assurance, EQ Four Element Calibration Beads (Fluidigm^®^, Cat. 201 078) were added to each sample to a final concentration of 10%. The acquisition was stopped when 0.5 million events were recorded. Data were analysed using Cytobank^®^. Standard VisNE configuration was used to perform the dimensional reduction analysis as previously reported.^46^

### Discriminant analysis and 3D plots

We used normal kernel density estimation (KDE), a non‐parametric discriminant analysis method, with the radius *R* = 0.2. A cross‐validation method was used for determining unbiased estimates of prior probabilities. The prior probabilities of healthy controls, patients in remission and patients with active disease were specified as 0.1, 0.2 and 0.7, respectively. The rate of error count estimates of the three groups was calculated for the combination of the frequencies of CCR7^+^, CD103^+^ and CXCR3^+^ Tregs and all the combinations of any two of the frequencies of the Treg subsets. Statistical significance was defined as *P* < 0.05. The discriminant analysis was conducted with SAS (Version 9.4 SAS Institute, Cary, NC). The 3D plots combining the frequency of CCR7, CD103 and CXCR3 Tregs were generated using *Partek Genomics Suite* software (Version 7.0, Partek Incorporated, St. Louis, MO, USA).

### Statistics

Data were analysed using Cytobank^®^ (Beckman Coulter) and SAS 9.4. Figures were made with GraphPad Prism 9 (La Jolla, CA, USA) and Partek Genomics suite 7 (St. Louis, MO, USA). The non‐parametric Mann–Whitney test was used to calculate significance. Bonferroni correction was applied for comparisons of more than two groups. *P*‐values of ≤ 0.05 and ≤ 0.01 were considered significant for two and three groups' comparisons respectively. *R*‐square and *P*‐values were reported for the correlation of Treg frequencies and BVAS. The non‐parametric Spearman's test was applied to determine the *R*‐square and *P*‐values of the correlation analysis.

## Author contributions

**Christian Agosto‐Burgos:** Conceptualization; data curation; formal analysis; investigation; methodology; project administration; validation; visualization; writing – original draft. **Eveline Y Wu:** Data curation; formal analysis. **Marie A Iannone:** Methodology; resources; visualization. **Yichun Hu:** Data curation; formal analysis; methodology; software. **Susan L Hogan:** Data curation; formal analysis; methodology. **Candace D Henderson:** Methodology; project administration; resources. **Kristin B Kennedy:** Methodology; project administration; resources. **Lauren Blazek:** Methodology; project administration; resources. **Carolina A Herrera:** Investigation; project administration. **Dominique Munson:** Project administration; resources. **Ronald J Falk:** Conceptualization; funding acquisition; project administration; supervision; writing – review and editing. **Dominic J Ciavatta:** Conceptualization; formal analysis; funding acquisition; investigation; methodology; project administration; supervision; writing – original draft; writing – review and editing. **Meghan E Free:** Conceptualization; formal analysis; funding acquisition; investigation; methodology; project administration; supervision; validation; visualization; writing – original draft; writing – review and editing.

## Conflict of interest

The authors declare no conflict of interest.

## Supporting information


Supporting information
