# Supplementary material for: The frequency of Treg subsets distinguishes disease activity in ANCA vasculitis
Source: Clin Transl Immunology. 2022 Nov 11;11(11):e1428. doi: 10.1002/cti2.1428 (PMC9652144; doi:10.1002/cti2.1428)
Supplement: Supplementary file 1 — Supporting information [file CTI2-11-e1428-s001.docx]

**Supplementary table 1:**

List of mass cytometry antibodies

| **Target** | **Metal** | **Clone** | **Vendor/Providor** |
| --- | --- | --- | --- |
| CD45 | 89Y | HI30 | Fluidigm |
| CD45 | 115In | HI30 | Lederer |
| CD49d | 141Pr | 9F10 | Fluidigm |
| CD278/ICOS | 143Nd | C398.4A | Fluidigm |
| CD4 | 145Nd | RPA-T4 | Fluidigm |
| HLA-DR | 147Sm | L243 | Lederer |
| CD194/CCR4 | 149Sm | L291H4 | Fluidigm |
| CD103 | 151Eu | Ber-ACT8 | Fluidigm |
| CD95/Fas | 152Sm | DX2 | Fluidigm |
| CD197 (CCR7) | 153Eu | G043H7 | RD Systems^†^ |
| CD3 | 154Sm | UCHT1 | Fluidigm |
| CD45RA | 155Gd | HI100 | Fluidigm |
| CD195 (CCR5) | 156Gd | NP-6G4 | Fluidigm |
| CD152 (CTLA-4) | 159Tb | 14D3 | Lederer |
| CD39 | 160Gd | A1 | Fluidigm |
| CD183 (CXCR3) | 162Dy | G025H7 | Lederer |
| FoxP3 | 165Ho | PCH101 | Lederer |
| CD223/LAG-3 | 166Er | 11C3C65 | Lederer |
| CD121a (IL-1b Receptor) | 167Er | MAB2692 | RD Systems^†^ |
| HELIOS | 168Er | 22F6 | Lederer |
| FoxP3d2 | 169Tm | 150D/E4 | eBioscience^‡^ |
| CD127 (IL-7Ra) | 171Yb | A019D5 | Lederer |
| CD25 (IL-2R) | 173Yb | 2A3 | Lederer |
| LAP | 174Yb | TW4-2F8 | Lederer |
| CD279 (PD-1) | 175Lu | EH12.2H7 | Fluidigm |
| CD196 (CCR6) | 176Yb | G034E3 | RD Systems^†^ |

^†^, conjugated in-house; ^‡^, conjugated by Fluidigm^©^

**Supplementary table 2:**

Error rate of discriminant analysis using normal-kernel density

|  | **Rate of Error Count Estimates for Status group** | | | | |
| --- | --- | --- | --- | --- | --- |
|  | **Variables** | **Act** | **HC** | **Rem** | **Total** |
| Training data | CCR7/CD103 | 0.1667 | 0.0000 | 0.0000 | 0.0550 |
|  | CCR7/CXCR3 | 0.0000 | 0.1667 | 0.0000 | 0.0556 |
|  | CD103/ CXCR3 | 0.0833 | 0.1667 | 0.0000 | 0.0833 |
|  | CCR7/CD103/CXCR3 | 0.0000 | 0.0000 | 0.0000 | 0.0000 |
| Test data | CCR7/CD103 | 0.5000 | 0.0000 | 1.0000 | 0.5000 |
|  | CCR7/CXCR3 | 0.5000 | 0.0000 | 0.6667 | 0.3889 |
|  | CD103/ CXCR3 | 0.0000 | 0.5000 | 1.0000 | 0.5000 |
|  | CCR7/CD103/CXCR3 | 0.0000 | 0.0000 | 0.6667 | 0.2222 |

**Supplementary Figure 1:**

Expression of the splice-variant FOXP3Δ2 in CD4^+^ T cells**.** VisNE from CD4^+^ T cells analysis revealing expression of FOXP3Δ2 within CD4^+^ T cells expressing intermediate or low levels of FOXP3 (non-Tregs).


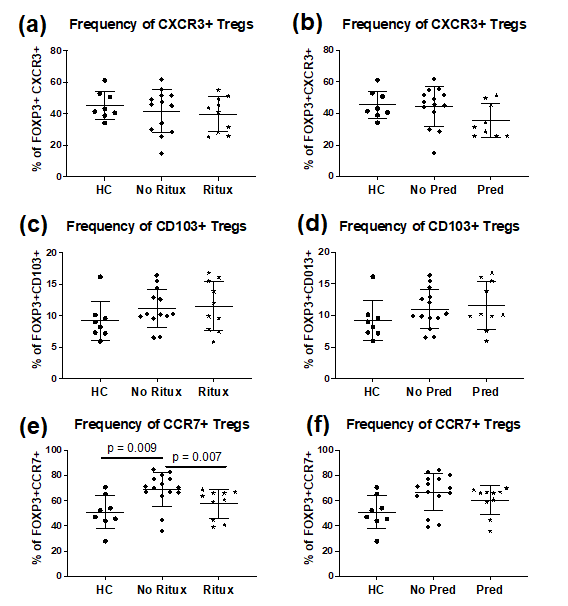


**Supplementary Figure 2:**

Frequency of CXCR3^+^, CD103^+^, and CCR7^+^ Treg subsets stratified by the administration of rituximab and prednisone. **(a, b):** Frequency of CXCR3^+^ Tregs stratified by the administration of rituximab **(a)** and prednisone **(b)**. **(c, d):** Frequency of CD103^+^ Tregs stratified by the administration of rituximab **(c)** and prednisone **(d)**. **(e, f):** Frequency of CCR7^+^ Tregs stratified by the administration of rituximab **(e)** and prednisone **(f)**.


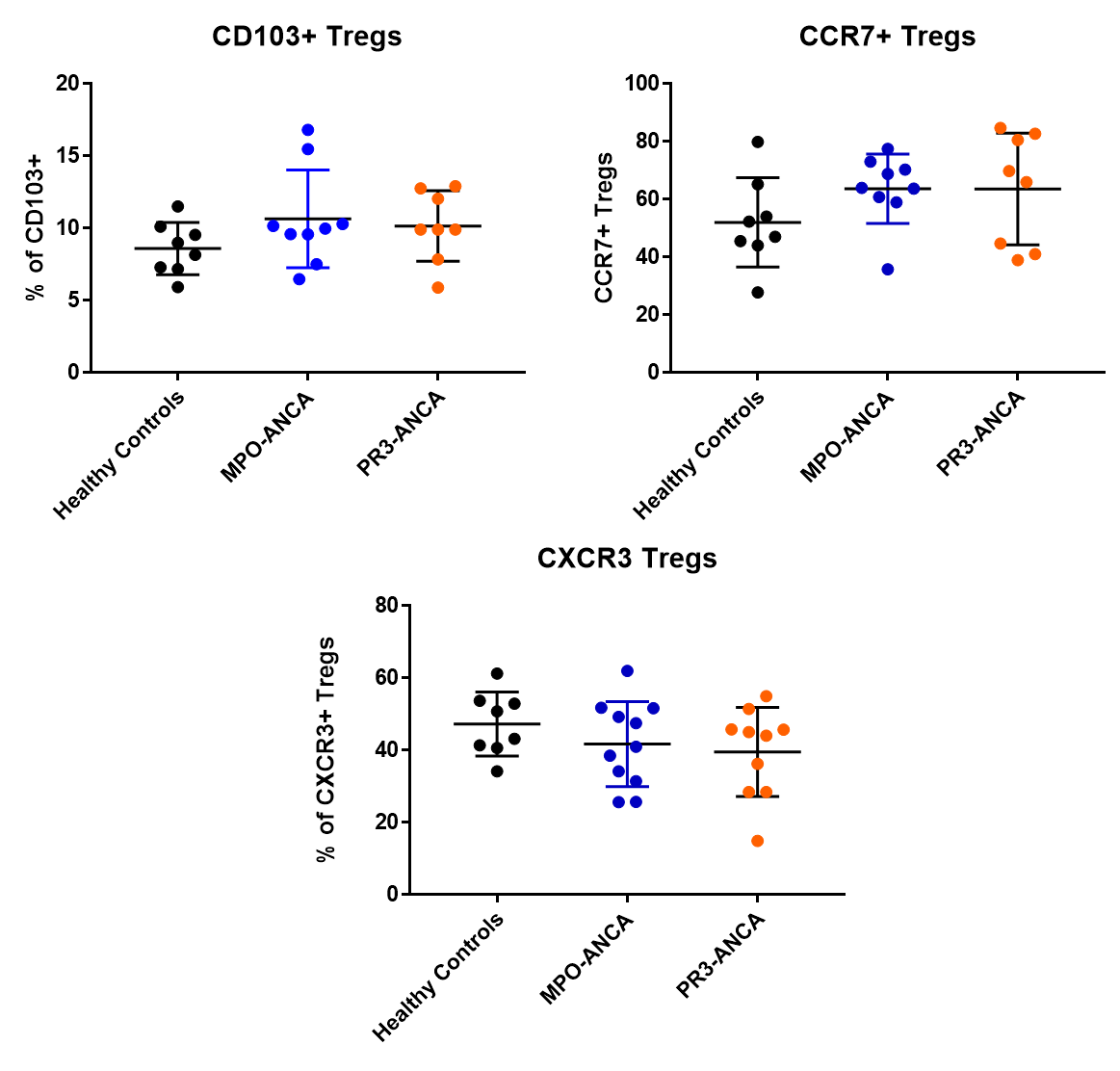


**Supplementary Figure 3:**

Frequency of CD103^+^, CCR7^+^, and CXCR3^+^ Treg subsets stratified by serotype (MPO-ANCA vs. PR3-ANCA). Serotype of ANCA vasculitis did not impact frequencies of Treg subsets.
